# Parameter Identifiability of Fundamental Pharmacodynamic Models

**DOI:** 10.3389/fphys.2016.00590

**Published:** 2016-12-05

**Authors:** David L. I. Janzén, Linnéa Bergenholm, Mats Jirstrand, Joanna Parkinson, James Yates, Neil D. Evans, Michael J. Chappell

**Affiliations:** ^1^Biomedical and Biological Systems Laboratory, School of Engineering, University of WarwickCoventry, UK; ^2^Drug Metabolism and Pharmacokinetics, Cardiovascular and Metabolic Diseases, iMED, AstraZenecaGothenburg, Sweden; ^3^Fraunhofer-Chalmers Centre, Chalmers Science ParkGothenburg, Sweden; ^4^Early Clinical Development, Quantitative Clinical Pharmacology, iMED, AstraZenecaGothenburg, Sweden; ^5^Oncology, iMED, AstraZenecaCambridge, UK

**Keywords:** structural identifiability, practical parameter identifiability, mixed effects models, pharmacodynamic models, fixed effects models

## Abstract

Issues of parameter identifiability of routinely used pharmacodynamics models are considered in this paper. The structural identifiability of 16 commonly applied pharmacodynamic model structures was analyzed analytically, using the input-output approach. Both fixed-effects versions (non-population, no between-subject variability) and mixed-effects versions (population, including between-subject variability) of each model structure were analyzed. All models were found to be structurally globally identifiable under conditions of fixing either one of two particular parameters. Furthermore, an example was constructed to illustrate the importance of sufficient data quality and show that structural identifiability is a prerequisite, but not a guarantee, for successful parameter estimation and practical parameter identifiability. This analysis was performed by generating artificial data of varying quality to a structurally identifiable model with known true parameter values, followed by re-estimation of the parameter values. In addition, to show the benefit of including structural identifiability as part of model development, a case study was performed applying an unidentifiable model to real experimental data. This case study shows how performing such an analysis prior to parameter estimation can improve the parameter estimation process and model performance. Finally, an unidentifiable model was fitted to simulated data using multiple initial parameter values, resulting in highly different estimated uncertainties. This example shows that although the standard errors of the parameter estimates often indicate a structural identifiability issue, reasonably “good” standard errors may sometimes mask unidentifiability issues.

## Introduction

Pharmacodynamic (PD) models quantify processes involved in drug action such as distribution to the effect site, receptor binding and signal transduction. PD models are valuable in making predictions of drug effects in un-tested scenarios such as outcomes across different populations or with new dosing schedules. Such predictions may not always be valid: In particular, there may be issues related to parameter identifiability. Within the concept of parameter identifiability, there are two distinct types: structural identifiability (Bellman and Åström, 1970) and practical identifiability (Raue et al., 2009).

As suggested by the name, structural identifiability concerns the inherent identifiability of the parameters in a model given its structure and observed outputs (Bellman and Åström, 1970). If a model is structurally unidentifiable, this means that at least one parameter can have any value without changing the model output (albeit with possible readjustment of remaining parameters). A well-known structurally unidentifiable problem is the linear model commonly used for estimating bioavailability *F* and volume of distribution *V* from plasma concentrations measured after oral drug administration, the most simple case being the one compartment PK model with first order absorption, where the plasma drug concentration *C* following a single dose is defined according to

(1) $$C(t)=\frac{F\cdot DOSE\cdot k_{a}}{V\left(k_{a}-k_{e}\right)}\left(e^{-k_{e}\cdot t}-e^{-k_{a}\cdot t}\right)$$

where *F* is the bioavailability of the drug, *DOSE* is the orally administered dose, *V* is the volume of distribution, *k*_*a*_ is the rate of absorption and *k*_*e*_ is the rate of elimination. It has been shown that only the fraction $\frac{F}{V}$ can be identified, and any estimate of *F* will therefore inversely correlate to *V* and both values will be biologically meaningless (Cheung et al., 2013). Importantly, predictions of *C*(*t*) are still valid as these depend on the identifiable fraction $\frac{F}{V}$. While structural identifiability is a property of the postulated model structure given a set of outputs, practical identifiability is related to the experimental data. In particular, it is a measure of the amount of information contained in the experimental data and how this information is translated to parameter uncertainty and subsequent prediction uncertainty.

**Figure 1:**
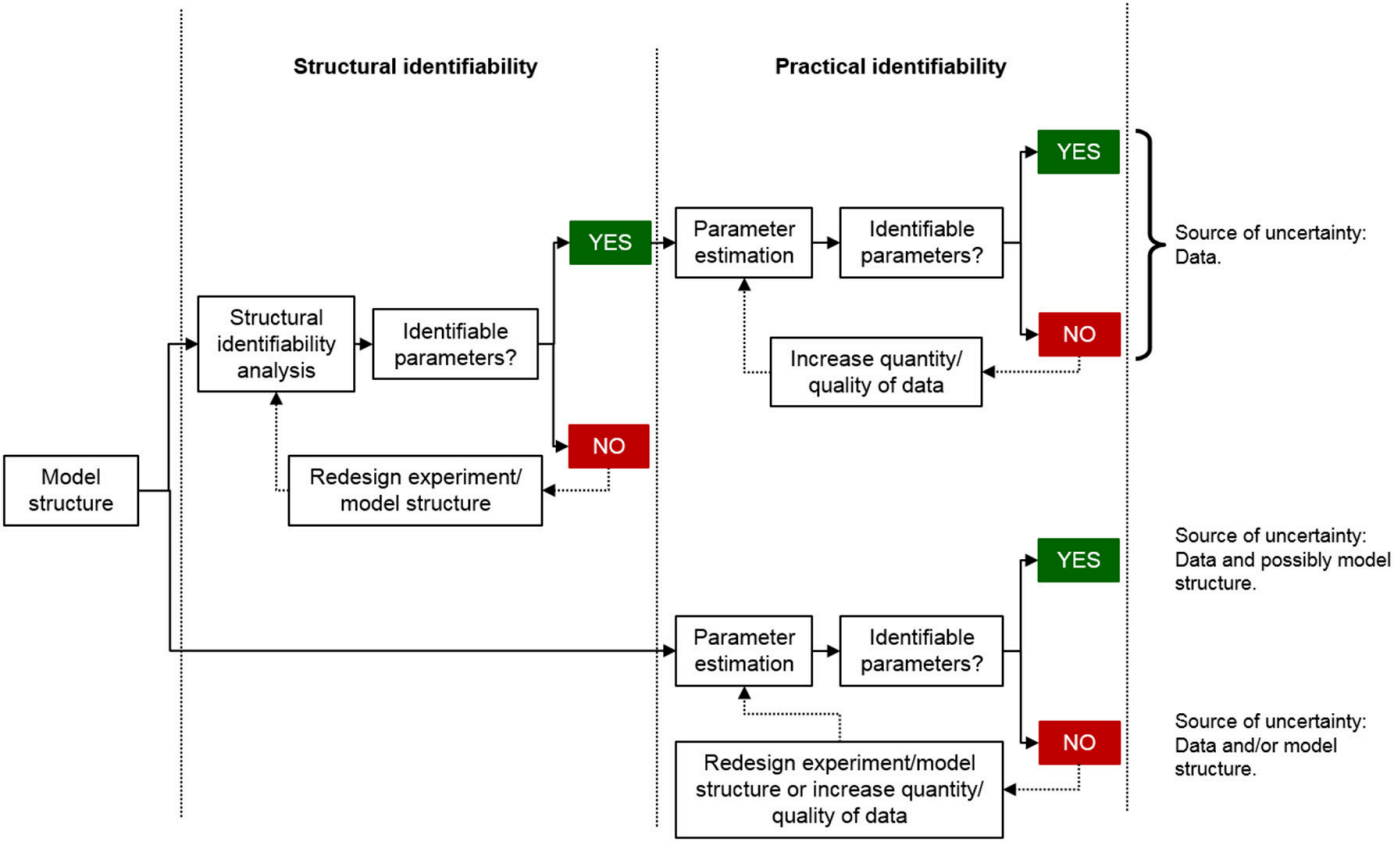
**Schematic comparing the model development process including or excluding a structural identifiability analysis**. If the structural identifiability of a model is known, the standard errors in the parameter estimates reflect the uncertainty in the data and and how well the model can describe them. However, if the structural identifiability is unknown, the standard errors in the parameter estimates may reflect both issues with the model structure and the data.

Parameter identifiability is unfortunately often only investigated and considered at the level of practical identifiability using more simple measurements such as standard errors or correlation matrices rather than more sophisticated approaches such as the profile likelihood approach (Raue et al., 2009). This is problematic for several reasons. The primary reason is that it cannot be guaranteed that the estimated parameter values are uniquely determined by just looking at the estimation results. In addition, if the structural identifiability of a model is unknown, it means that the source of uncertainty in the parameter estimates may be either due to the experimental data, the model structure, or both (Figure 1). Thus, increasing the quality of the data may or may not improve the precision of the parameter estimates. However, if structural identifiability analysis has concluded that the model is identifiable, the uncertainty in the model parameters is directly linked to the quality of the data and how well the model can describe them. In this scenario, the uncertainty of the model parameters can be improved by increasing the quality of the data. However, there will always be uncertainties in the parameter estimates even if the model is structurally identifiable and the quality and quantity of the experimental data are relatively high. An approach to further strengthen the plausibility of the model predictions under such conditions is to divide the experimental data into two parts: data used for parameter estimation and data used for model validation, i.e., by estimating the unknown parameters using a subset of the experimental data and using the resulting estimates to predict the validation data.

To further exemplify the importance of structural identifiability, consider the two following biological examples. In Evans et al. (2004), a model which aims to describe the activity of an anti-cancer agent named topotecan and its delivery to nuclear target DNA is presented. Prior to parameter estimation it was found that a subset of the model parameters was unidentifiable but if additional experimental measurements were made, in this case determining volume ratios, then the model would become structurally identifiable. In Evans et al. (2001), a parent-metabolite model for ivabradine is considered. The model was shown to be structurally unidentifiable with either intravenous, oral or combined intravenous and oral administration. It was also shown that by either fixing the volume parameter for the central compartment, or with a particular simplification of the model structure, then the model becomes identifiable for the given observations. If a formal structural identifiability analysis had not been performed then these two research projects would have most likely continued without these insights with the potential risk of missleading outcomes.

While PD models are highly diverse, many basic processes involved in drug action are similar across drugs and systems, such as distribution from the plasma to the target tissues, interaction with a target such as receptor binding or altered rates of production or loss of a target. These general processes have been described using semi-mechanistic models. For example, the effect compartment model (Sheiner et al., 1979) has been used to describe short delays in drug action due to distributional delays using a hypothetical “effect compartment.” Similarly, receptor binding models (Danhof et al., 2007; Gabrielsson et al., 2011), turnover models (Gabrielsson et al., 2011) and the operational model (Black and Leff, 1983; Danhof et al., 2007) have been used to describe the processes of drug binding and signaling. However, despite frequent use, relatively few PD models have been analyzed from a structural identifiability perspective. An example of a published structural identifiability analysis is for an approximation of the receptor binding model. Receptor binding often occurs over very fast timescales relative to the PK, and sometimes also with respect to the effects elicited by the receptor once bound. In such cases, the receptor binding model may be approximated by a quasi- or pseudo-steady state approximation. When using such an approximation, it has been shown that the individual on and off rates of drug binding to the receptor cannot be uniquely identified (Chappell, 1996). Another example is the target-mediated drug disposition model (Mager and Jusko, 2001) applicable to the modeling of biologics, which has been shown to be structurally identifiable (Eudy et al., 2015). However, the identifiability of the effect compartment model and the operational model have, to our knowledge, not previously been analyzed. Furthermore, mixed effects (“population”) models are often used to account for and quantify known sources of variability in data sets, such as between-subject variability (BSV). Such models are combined structural and statistical models, with additional statistical parameters describing the variance of a postulated distribution of the model parameter values across e.g., subjects. The structural identifiability of mixed effects models describing BSV has not previously been analyzed.

The primary goal of this paper is to illustrate the concept and importance of parameter identifiability, both from a structural and practical perspective. Structural identifiability analysis is performed on a family of 16 commonly used PD models to serve as a database for modelers in the pharmaceutical domain. Both fixed-effects models and the corresponding mixed-effects (population) models are analyzed. Pharmacodynamic models describing combinations of none to three different mechanisms of delays in drug action are analyzed: (i) delays in drug distribution to the site of action applying the effect compartment model, (ii) delays in signal transduction, build-up or loss of effect applying turnover models and (iii) delays due to slow dissociation to the target applying receptor binding models. These and similar models are extensively used within mechanism-based PD models in pharmaceutical research (Ploeger et al., 2009; Peletier and Gabrielsson, 2012). In addition, the problem of structural identifiability and its relation to practical identifiability will be illustrated through a set of examples using both simulated data and real experimental data.

## Methods

Structural identifiability analysis has been performed on all models written in state-space form. A fixed-effects state-space model is written on the following form

(2) $$\dot{x}(t)=f(x(t),u(t),\theta ),\;x(t_{0})=x_{0}$$

(3) y(t)=h(x(t),u(t),θ)

where ***x***(*t*) ∈ ℝ^*n*^ is the state (e.g., plasma concentration of the drug, bound and unbound receptors etc.) ***u***(*t*) ∈ ℝ^*q*^ is the input (IV bolus, IV infusion etc.), **θ** ∈ ℝ^*p*^ is the vector of model parameters (e.g., clearance rate, maximum saturation, etc.), ***y***(*t*) ∈ ℝ^*m*^ is the output (measurement of plasma concentration, drug effects) and ***f*** and ***h*** are smooth functions as *C*^∞^ with respect to the functional arguments.

A mixed-effects model is written on one of the forms

(4) $${\dot{x}}_{i}(t)=f(x_{i}(t),u_{i}(t),\phi_{i})\;x_{i}(t_{0})=x_{0}(\phi_{i})$$

(5) $$y_{i}(t)=h(x_{i}(t),u_{i}(t),\phi_{i})$$

where **ϕ**_*i*_ = *g*(**θ**, **η**_*i*_, ***C***_*i*_) are the parameters for the *i*:th subject, **η**_*i*_ ~ *N*(**0**, **Ω**) are the random effects variables where **Ω** is the variance-covariance matrix of the random effects **η**_*i*_, **θ** are the population parameters and ***C***_*i*_ are the covariates for the different subjects in the population.

### Structural identifiability: definition

As mentioned in the introduction, structural identifiability is a theoretical concept with direct practical relevance. This is because if a model is structurally unidentifiable, some of the model parameters may take on arbitrary numerical values while the model may still describe the experimental data equally well. In a numerical structural identifiability analysis different numerical values are sought that will result in identical model responses. In an analytical structural identifiability analysis, more general conclusions can often be drawn since in such as analysis symbolic relationsships between the model parameters can be derived allowing for suggestions of reparametrization and/or additional measurements required to render an unidentifable model to become identifiable. Since different values of the unidentifiable parameters result in identical responses or predictions any subsequent biological interpretations of the estimates of those unidentifiable parameters (e.g., clearance, *IC*_50_) are effectively meaningless in a biological context. It is because of this that structural identifiability is often referred to as a prerequisite to successful parameter estimation. In other words, if a structural identifiability analysis (in which perfect experimental conditions e.g., noise-free and continuous measurements, are assumed) has shown that some of the model parameters can not be determined, it follows directly that these parameters can never be determined in the less ideal case, i.e., under real experimental conditions for discrete measurements with noise present.

To define exactly what is meant by structural identifiability there now follows a more rigorous mathematical definition of the concept in the context of fixed-effects models.

Let the generic parameter vector **θ** belong to a feasible parameter space **Θ**, i.e., **θ** ∈ Θ. Let *y*(*t*, **θ**) be the output function from the state-space model. Further, consider a parameter vector $\bar{\theta }$ where $\text{y}\left(t,\theta \right)=\text{y}\left(t,\bar{\theta }\right)$ for all *t*. If this equality, in a neighborhood ***N*** ⊂ Θ of **θ**, implies that $\theta =\bar{\theta }$ then the model is *structurally locally identifiable*. If ***N*** = Θ then the model is *structurally globally identifiable*. If a model is structurally unidentifiable, then every neighborhood of **θ** contains a $\bar{\theta }\neq \theta$ such that $y\left(t,\theta \right)=y\left(t,\bar{\theta }\right)$ for all *t*.

Since the mixed-effects models to be considered in this paper are also analyzed from a structural identifiability perspective it must first be defined what is meant by the identifiability of such models. Since mixed-effects models yield individual predictions, in contrast to single predictions in the fixed-effects case, the previous definition is not immediately applicable to mixed-effects models. Instead, a generalized version of the definition of structural identifiability is used. In this new definition, first presented in Janzén et al. (2016), a model is defined to be structurally identifiable if the distribution of the output from the model determines both the structural and statistical parameters, i.e., the parameters in the vector **θ** and the variance parameters in **Ω** denoting the variance of the random effects **η** respectively. Now follows a more rigorous definition of structural identifiable for mixed-effects models.

Let *p*(***y***_{**θ**, **Ω**}_, *t*) denote the distribution of the output signals ***y*** at time *t*. Let the generic parameter vector and matrix {**θ**, **Ω**} belong to a feasible parameter space {**θ**, **Ω**} ∈ **Θ**, and consider the following two sets of parameters {**θ**, **Ω**} and $\left\{\bar{\theta },\bar{\Omega }\right\}$. If $p\left({\text{y}}_{\left\{\theta ,\Omega \right\}},t\right)=p\left({\text{y}}_{\left\{\bar{\theta },\bar{\Omega }\right\}},t\right)$ for all *t* implies that $\left\{\theta ,\Omega \right\}=\left\{\bar{\theta },\bar{\Omega }\right\}$ in a neighborhood *N* ⊂ Θ then the model is *structurally locally identifiable*, and if *N* = Θ the model is *structurally globally identifiable*. For a *structurally unidentifiable* parameter, θ_*i*_, or ω_*i*_ ∈ **Ω**, every neighborhood *N* around θ_*i*_, or ω_*i*_, has a parameter vector/matrix $\bar{\theta }$, or $\bar{\Omega }$, where $\theta_{i}\neq {\bar{\theta }}_{i}$, or $\omega_{i}\neq {\bar{\omega }}_{i}$, give rise to the same distribution of identical input-output relations.

### Investigated model structures

**Figure 2:**
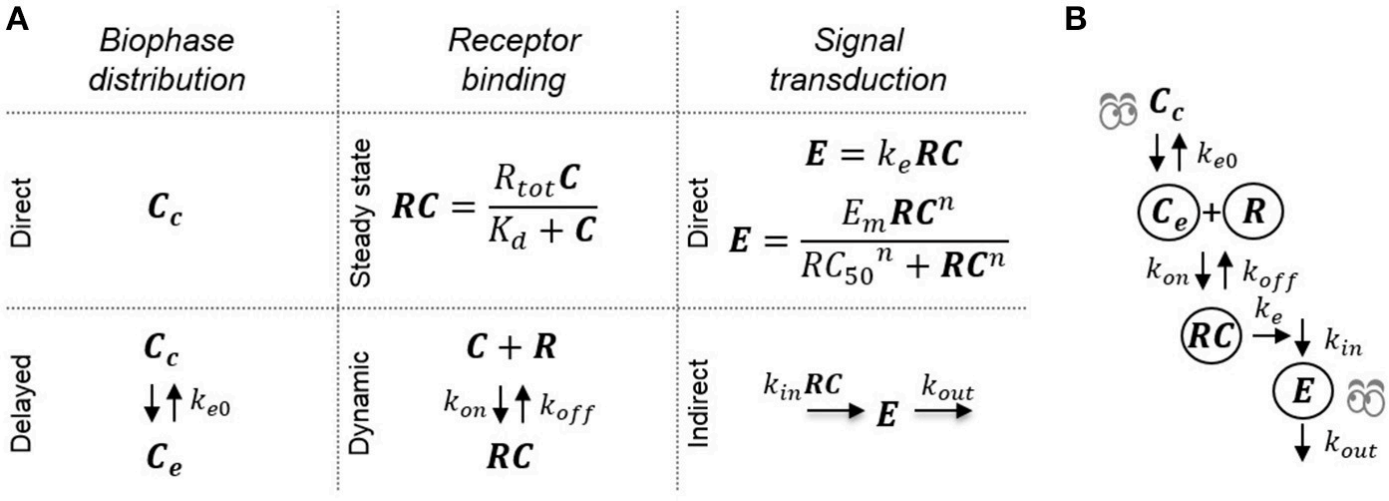
**Schematic of the investigated pharmacodynamic models**. **(A)** The 16 investigated models are constructed by combining the following submodels: Direct or delayed biophase concentration through distribution to a hypothetical effect compartment, dynamic or direct receptor binding using the steady-state approximation and direct proportional or sigmoid signal transduction or delayed signal transduction applying a turnover model. **(B)** Example of a full model where all three processes are assumed to be dynamic and cause delay between plasma concentration and drug effect.

The model structures investigated to determine structural identifiability were all combinations of sub-models representing receptor binding, a hypothetical effect compartment and direct or indirect transduction (see Figure 2). In total, 16 different model structures were investigated (Table 1). Both fixed-effects and mixed-effects versions of each model were analyzed from a structural identifiability perspective.

**Table 1:** **Summary of the 16 PD fixed effects and mixed effects models for which the structural identifiability was investigated**.

***N***	**Model equations**	**I/O**	**ICs**	**Fixed effects models**	**Mixed effects models**	
				**Fixed effect parameters**	**Fixed effect parameters^a^**	**Random effect parameters^b^**
1	$E=k_{e}\frac{R_{tot}C_{p}}{K_{d}+C}$	*C*_*p*_/*E*		*R*_*tot*_, *k*_*e*_, *K*_*d*_	*k*_*e*_, *K*_*d*_	η_*ke*_, η_*Kd*_
2	$E=\frac{E_{m}{\left(R_{tot}C_{p}\right)}^{n}}{{\left(K_{d}+C_{p}\right)}^{n}RC_{50}^{n}+{\left(R_{tot}C_{p}\right)}^{n}}$	*C*_*p*_/*E*		*R*_*tot*_, *E*_*m*_, *RC*_50_, *n, K*_*d*_	*E*_*m*_, *RC*_50_, *n, K*_*d*_	η_*Em*_, η_*RC*50_, η_*n*_, η_*Kd*_
3	$Ė=k_{in}\left(1+k_{e}\frac{R_{tot}C_{p}}{K_{d}+C_{p}}\right)-k_{out}E$	*C*_*p*_/*E*	*E*(0) = *k*_*out*_/*k*_*in*_	*R*_*tot*_, *k*_*in*_, *k*_*out*_, *k*_*e*_, *K*_*d*_	*k*_*in*_, *k*_*out*_, *k*_*e*_, *K*_*d*_	η_*kin*_, η_*kout*_, η_*ke*_, η_*Kd*_
4	$Ė=k_{in}-k_{out}\left(1+k_{e}\frac{R_{tot}C_{p}}{K_{d}+C_{p}}\right)E$	*C*_*p*_/*E*	*E*(0) = *k*_*out*_/*k*_*in*_	*R*_*tot*_, *k*_*in*_, *k*_*out*_, *k*_*e*_, *K*_*d*_	*k*_*in*_, *k*_*out*_, *k*_*e*_, *K*_*d*_	η_*kin*_, η_*kout*_, η_*ke*_, η_*Kd*_
5	$\dot{RC}=k_{on}\left(R_{tot}-RC\right)C_{p}-k_{off}RC$	*C*_*p*_/*E*	*RC*(0) = 0	*R*_*tot*_, *k*_*on*_, *k*_*off*_, *k*_*e*_	*k*_*on*_, *k*_*off*_, *k*_*e*_	η_*kon*_, η_*koff*_, η_*ke*_
	*E* = *k*_*e*_*RC*					
6	$\dot{RC}=k_{on}\left(R_{tot}-RC\right)C_{p}-k_{off}RC$	*C*_*p*_/*E*	*RC*(0) = 0	*R*_*tot*_, *k*_*on*_, *k*_*off*_, *E*_*m*_, *RC*_50_, *n*	*k*_*on*_, *k*_*off*_, *E*_*m*_, *RC*_50_, *n*	η_*RC*50_, η_*kon*_, η_*koff*_, η_*Em*_, η_*n*_
	$E=\frac{E_{m}RC^{n}}{RC_{50}^{n}+RC^{n}}$					
7	$\dot{RC}=k_{on}\left(R_{tot}-RC\right)C_{p}-k_{off}RC$	*C*_*p*_/*E*	*RC*(0) = 0	*R*_*tot*_, *k*_*on*_, *k*_*off*_, *k*_*in*_, *k*_*out*_, *k*_*e*_	*k*_*on*_, *k*_*off*_, *k*_*in*_, *k*_*out*_, *k*_*e*_	η_*kon*_, η_*koff*_, η_*kin*_, η_*kout*_, η_*ke*_
	Ė = *k*_*in*_(1+*k*_*e*_*RC*)−*k*_*out*_*E*		*E*(0) = *k*_*out*_/*k*_*in*_			
8	$\dot{RC}=k_{on}\left(R_{tot}-RC\right)C_{p}-k_{off}RC$	*C*_*p*_/*E*	*RC*(0) = 0	*R*_*tot*_, *k*_*on*_, *k*_*off*_, *k*_*in*_, *k*_*out*_, *k*_*e*_	*k*_*on*_, *k*_*off*_, *k*_*in*_, *k*_*out*_, *k*_*e*_	η_*kon*_, η_*koff*_, η_*kin*_, η_*kout*_, η_*ke*_
	Ė = *k*_*in*_ − *k*_*out*_(1+*k*_*e*_*RC*)*E*		*E*(0) = *k*_*out*_/*k*_*in*_			
9	$\dot{C_{e}}=k_{e0}*\left(C_{p}-C_{e}\right)$	*C*_*p*_/*E*	*C*_*e*_(0) = 0	*k*_*e*0_, *R*_*tot*_, *k*_*e*_, *K*_*d*_	*k*_*e*0_, *k*_*e*_, *K*_*d*_	η_*ke*0_, η_*ke*_, η_*Kd*_
	$E=k_{e}\frac{R_{tot}C_{e}}{K_{d}+C_{e}}$					
10	$\dot{C_{e}}=k_{e0}*\left(C_{p}-C_{e}\right)$	*C*_*p*_/*E*	*C*_*e*_(0) = 0	*k*_*e*0_, *R*_*tot*_, *E*_*m*_, *RC*_50_, *n, K*_*d*_	*k*_*e*0_, *E*_*m*_, *RC*_50_, *n, K*_*d*_	η_*RC*50_, η_*ke*0_, η_*Em*_, η_*n*_, η_*Kd*_
	$E=\frac{E_{m}{\left(R_{tot}C_{e}\right)}^{n}}{{\left(K_{d}+C_{e}\right)}^{n}RC_{50}^{n}+{\left(R_{tot}C_{e}\right)}^{n}}$					
11	$\dot{C_{e}}=k_{e0}*\left(C_{p}-C_{e}\right)$	*C*_*p*_/*E*	*C*_*e*_(0) = 0	*k*_*e*0_, *R*_*tot*_, *k*_*in*_, *k*_*out*_, *k*_*e*_, *K*_*d*_	*k*_*e*0_, *k*_*in*_, *k*_*out*_, *k*_*e*_, *K*_*d*_	η_*ke*0_, η_*kin*_, η_*kout*_, η_*ke*_, η_*Kd*_
	$Ė=k_{in}\left(1+k_{e}\frac{R_{tot}C_{e}}{K_{d}+C_{e}}\right)-k_{out}E$		*E*(0) = *k*_*out*_/*k*_*in*_			
12	$\dot{C_{e}}=k_{e0}*\left(C_{p}-C_{e}\right)$	*C*_*p*_/*E*	*C*_*e*_(0) = 0	*k*_*e*0_, *R*_*tot*_, *k*_*in*_, *k*_*out*_, *k*_*e*_, *K*_*d*_	*k*_*e*0_, *k*_*in*_, *k*_*out*_, *k*_*e*_, *K*_*d*_	η_*ke*0_, η_*kin*_, η_*kout*_, η_*ke*_, η_*Kd*_
	$Ė=k_{in}-k_{out}\left(1+k_{e}\frac{R_{tot}C_{e}}{K_{d}+C}\right)E$		*E*(0) = *k*_*out*_/*k*_*in*_			
13	$\dot{C_{e}}=k_{e0}*\left(C_{p}-C_{e}\right)$	*C*_*p*_/*E*	*C*_*e*_(0) = 0	*k*_*e*0_, *R*_*tot*_, *k*_*on*_, *k*_*off*_, *k*_*e*_	*k*_*e*0_, *k*_*on*_, *k*_*off*_, *k*_*e*_	η_*ke*0_, η_*kon*_, η_*koff*_, η_*ke*_
	$\dot{RC}=k_{on}\left(R_{tot}-RC\right)C_{e}-k_{off}RC$		*RC*(0) = 0			
	*E* = *k*_*e*_*RC*					
14	$\dot{C_{e}}=k_{e0}*\left(C_{p}-C_{e}\right)$	*C*_*p*_/*E*	*C*_*e*_(0) = 0	*k*_*e*0_, *R*_*tot*_, *RC*_50_, *k*_*on*_, *k*_*off*_, *E*_*m*_, *n*	*k*_*e*0_, *RC*_50_, *k*_*on*_, *k*_*off*_, *E*_*m*_, *n*	η_*ke*0_, η_*RC*50_, η_*kon*_, η_*koff*_, η_*Em*_, η_*n*_
	$\dot{RC}=k_{on}\left(R_{tot}-RC\right)C_{e}-k_{off}RC$		*RC*(0) = 0			
	$E=\frac{E_{m}RC^{n}}{RC_{50}^{n}+RC^{n}}$					
15	$\dot{C_{e}}=k_{e0}*\left(C_{p}-C_{e}\right)$	*C*_*p*_/*E*	*C*_*e*_(0) = 0	*k*_*e*0_, *R*_*tot*_, *k*_*on*_, *k*_*off*_, *k*_*in*_, *k*_*out*_, *k*_*e*_	*k*_*e*0_, *k*_*on*_, *k*_*off*_, *k*_*in*_, *k*_*out*_, *k*_*e*_	η_*ke*0_, η_*kon*_, η_*koff*_, η_*kin*_, η_*kout*_, η_*ke*_
	$\dot{RC}=k_{on}\left(R_{tot}-RC\right)C_{e}-k_{off}RC$		*RC*(0) = 0			
	Ė = *k*_*in*_(1+*k*_*e*_*RC*)−*k*_*out*_*E*		*E*(0) = *k*_*out*_/*k*_*in*_			
16	$\dot{C_{e}}=k_{e0}*\left(C_{p}-C_{e}\right)$	*C*_*p*_/*E*	*C*_*e*_(0) = 0	*k*_*e*0_, *R*_*tot*_, *k*_*on*_, *k*_*off*_, *k*_*in*_, *k*_*out*_, *k*_*e*_	*k*_*e*0_, *k*_*on*_, *k*_*off*_, *k*_*in*_, *k*_*out*_, *k*_*e*_	η_*ke*0_, η_*kon*_, η_*koff*_, η_*kin*_, η_*kout*_, η_*ke*_
	$\dot{RC}=k_{on}\left(R_{tot}-RC\right)C_{e}-k_{off}RC$		*RC*(0) = 0			
	Ė = *k*_*in*_ − *k*_*out*_(1+*k*_*e*_*RC*)*E*		*E*(0) = *k*_*out*_/*k*_*in*_			

N, Model number; I/O, Model inputs/outputs; ICs, Initial conditions.^a^R_tot_ was fixed at 100 when analysing the mixed effects models.^b^Each mixed-effects model was assumed to have a diagonal covariance matrix **Ω** with lognormally distributed random effects.

#### Structural identifiability: example

To exemplify the structural identifiability analysis, a summary of the analysis of the structural identifiability of Model 13 (Table 1) is provided. This model is a dynamic receptor binding model with an effect compartment and linear transduction. The details of the structural identifiability analysis for this model is available in the Supplementary Materials. The mathematical model has the following structure

(6) $$\begin{array}{l} \;{\dot{C}}_{e}=k_{e0}(C_{p}-C_{e})\\ \dot{RC}=k_{on}(R_{tot}-RC)C_{e}-k_{off}RC\\ \;E=k_{e}RC \end{array}$$

with the unknown parameter vector **θ** = (*k*_*e*0_, *k*_*e*_, *k*_*on*_, *k*_*off*_) and where *C*_*p*_ is the concentration in the blood plasma and is in this case a known input signal, *C*_*e*_ is a state representing the concentration in the hypothetical effect compartment, *RC* is the receptor complex, *E* is the observed effect and *R*_*tot*_ representing the percentage of total number of receptors, which is fixed at 100%.

The approach chosen to study structural identifiability here is the input-output approach, for which details can be found in Bearup et al. (2013). A general outline of the method is given here followed by an example of how a structural identifiability analysis is performed.

The input-output approach used in this paper was chosen for three reasons. The first reason was because the input-output approach can be used to show whether a model is globally or locally identifiable, or unidentifiable. Some of the other methods that are available for performing a structural identifiability analysis can only be used to show whether a model is at least locally identifiable or unidentifiable. The second reason was that there is a direct extension from non-population (fixed-effects) models to population (mixed-effects) models when it comes to structural identifiability analysis using the input-output approach as will be explained further below. The third reason is because the method is applicable to both linear and nonlinear models.

The main idea behind the input-output form approach is to transform the model to a form from which the identifiability problem can more easily be studied. This is performed by iteratively computing higher order time derivatives of the output function and using subsequent substitution to eliminate all state variables in order to express the system as a monomial solely in terms of the output functions(s) and its (their) derivatives. As the assumption of perfect experimental conditions is made, it follows that the output function and its higher order derivatives are assumed to be known. In other words, a model rewritten on an input-output form is a single equation with the output function and its higher order derivatives being known and the model parameters (that enters as the monomial coefficients) being unknown. Determining whether a model is structurally identifiable or otherwise is then a case of showing whether the resultant input-output equation has a single, finite, or an infinite number of solutions for the parameters in the coefficient expressions.

By iteratively differentiating the output signal and eliminating the state variables the model can be rewritten in the following input-output form

(7) $$\begin{array}{l} -R_{tot}{}^{2}C_{p}k_{e}^{2}k_{e0}k_{on}-2R_{tot}C_{p}k_{e}k_{e0}k_{on}E+R_{tot}k_{e}k_{e0}k_{off}E-\\ C_{p}k_{e0}k_{on}E^{2}+R_{tot}k_{e}k_{e0}\dot{E}+R_{tot}k_{e}k_{off}\dot{E}+k_{e0}k_{off}E^{2}+\\ R_{tot}k_{e}\ddot{E}+k_{e0}E\dot{E}+E\ddot{E}-{\dot{E}}^{2}=0. \end{array}$$

The structural identifiability of a model can then be studied by considering the coefficients in the input-output form of the model. Introducing an alternative parameter vector $\bar{\theta }$ and collecting the coefficients in the input-output form as

(8) $$\sum_{k\;=\;1}^{l}c_{k}(\theta ,\bar{\theta })\phi_{k}(E(t,\theta ),\dot{E}(t,\theta ),\ddot{E}(t,\theta ),\ldots )=0$$

permits determination of whether the model is structurally identifiable or otherwise, given that the ϕ_*k*_(·) are linearly independent. The analysis shows that $\theta =\bar{\theta }$, meaning that model 13 is structurally globally identifiable (details are given in the Supplementary Materials).

Similarly, a mixed-effects version of the model can be studied by using the coefficients in the input-output relation. As outlined and discussed in detail in Janzén et al. (Under review), since individual estimates are obtained in a mixed-effects model a distribution, assuming an infinite number of subjects (i.e., ideal experimental conditions in a mixed-effects context), of *c*_*k*_(**θ**) is in turn obtained. This distribution is directly linked to the distribution of the output functions. By introducing the random effects on the coefficients from the input-output form, according to the statistical sub-model, functions of random variables are derived. By studying whether the distributions of the generated functions of random variables determine both the fixed effects and the random effects related parameters, conclusions regarding whether the mixed-effects model is structurally identifiable or otherwise can be made. The mixed-effects version of model 13 with lognormally distributed random effects on all model parameters with a diagonal covariance matrix is also structurally globally identifiable. This follows from the fact that the structural model has been shown to be structurally globally identifiable (detail in the Supplementary Material) and the statistical parameters are uniquely determined by the lognormal distribution.

It is worth mentioning that structural identifiability analysis using analytical techniques such as the input-output approach may encounter certain limitations in terms of model size and complexity. In general, the more complex a model is in terms of the state-space dimensions and number of unknown parameters, then the more computationally demanding the subsequent analysis may become. If an analytical approach is not possible due to symbolic computational intractability then a hybrid symbolic/numerical analysis approach is an alternative, see the profile likelihood approach (Raue et al., 2009) or the Exact Arithmetic Rank approach (Karlsson et al., 2012). For an extensive comparison between the profile likelihood approach, the Exact arithmetic Rank approach and a differential algebra approach implemented in a software called DAISY, see Raue et al. (2014).

### Practical identifiability

**Figure 3:**
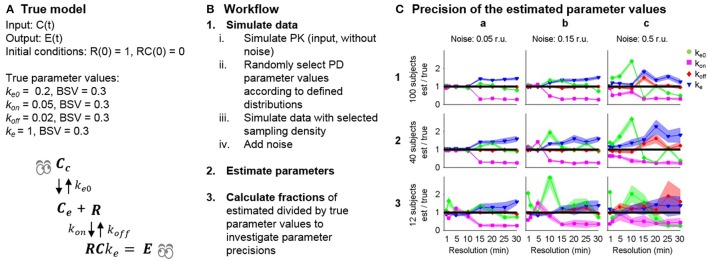
**(A)** Model 13 and the selected true parameter values. BSV is between-subject variability. **(B)** Workflow for the simulation study. **(C)** Accuracy of the typical parameter estimates for the combined effect compartment/dynamic receptor model fitted to simulated data. The accuracy of each parameter (y-axis: estimated/true parameter value, line of unity marked by black line) and its uncertainty (normalized standard error, filled lighter area) is given at each data resolution level (x-axis: time between samples increasing from 1, 2, 5, 10, 15, 20, 25 to 30 min) for simulated data for 100, 40 and 12 subjects (row 1, 2, 3) adding additive noise with standard deviations 0.05, 0.15, and 0.5 response units (r.u, column a, b, c) respectively.

Once the structural identifiability of the postulated model has been determined, parameter estimation can be performed. As with the structural identifiability example, Model 13 (Table 1) was selected for the simulation study to investigate the influence of varying data quality on the practical identifiability of the parameters. This model includes two different sources of delay, one from distribution to the effect site, where the rate is controlled by the parameter *k*_*e*0_, and also through slow receptor dynamics, where the off-rate is controlled by the parameter *k*_*off*_. The possibility to distinguish the two different delays in practice under varying data quality was investigated in a simulation study. *R*_*tot*_ was fixed to 1 following the results of the structural identifiability analysis to ensure the structural identifiability of the model. True parameter values were assigned to each model parameter: *k*_*e*_ = 1, *k*_*e*0_ = 0.2, *k*_*off*_ = 0.02 and *k*_*on*_ = 0.05 amounts per minute. All parameters were assumed to vary between subjects following a log-normal distribution as this ensures positive rates for all subjects, with standard deviation σ = 0.3 amounts per minute to represent differences in a population. The model is summarized in Figure 3A.

The simulation study was performed in MATLAB 2013b (The MathWorks, Inc., 2016) and Monolix 4.3.2 (Lixoft, 2012) as outlined in Figure 3B. (1) PK data were simulated without variability or noise, applying an intravenous bolus dose of 20 mg/kg to a hypothetical typical individual with volume of distribution 1 and rate of elimination 0.2 mg/kg. (2) Model 13 with the selected “true” parameter values was used to simulate data sets of varying size and quality. Three factors were changed that influence the information available in the data: (i) different sampling densities Δ*t* = 1, 2, 5, 10, 15, 20, 25, 30 min. (ii) different additive noise levels σ = 0.05, 0.15, 0.5 response units and (iii) different numbers of subjects *n* = 100, 40, 12. (3) Parameters were estimated using each simulated data set, with the following initial guess selected for the optimization algorithm: *k*_*e*_ = 1, *k*_*e*0_ = 0.1, *k*_*off*_ = 0.01 and *k*_*on*_ = 0.01 units per minute for the structural parameters and 0.3 units per minute for the standard deviations. (4) The ratio between the final parameter estimates and the true parameter values were calculated and compared for the typical parameters to investigate the effects of varying sampling frequency, noise levels and number of subjects on parameter accuracy.

## Results

### Structural identifiability analysis

The results of the structural identifiability analysis applying the input-output approach are summarized in Table 2, including the structural identifiability results and the reparameterization solutions to achieve structurally identifiable models.

**Table 2:** **Results of the structural identifiability analysis of the mixed-effects models 1–16 in Table 1**.

**Model description**		**Structural identifiability results**		
		**Fixed effects**		**Random effects**^a^
***N***	**Distr**. | **Binding** | **Transd**.	**SU parameters**	**SI parameters & combinations**	**SI parameters**
1	Direct | SS | Linear	*R*_*tot*_, *k*_*e*_	*R*_*tot*_*k*_*e*_, *K*_*d*_	η_*ke*_, η_*Kd*_
2	Direct | SS | Sigmoid	*R*_*tot*_, *RC*_50_	*R*_*tot*_/*RC*_50_, *k*_*e*_, *K*_*d*_, *n*	η_*RC*50_, η_*ke*_, η_*Kd*_, η_*n*_
3	Direct | SS | Indirect	*R*_*tot*_, *k*_*e*_	*R*_*tot*_*k*_*e*_, *k*_*in*_, *k*_*out*_, *K*_*d*_	η_*kin*_, η_*kout*_, η_*ke*_, η_*Kd*_
4	Direct | SS | Indirect	*R*_*tot*_, *k*_*e*_	*R*_*tot*_*k*_*e*_, *k*_*in*_, *k*_*out*_, *K*_*d*_	η_*kin*_, η_*kout*_, η_*ke*_, η_*Kd*_
5	Direct | Dynamic | Linear	*R*_*tot*_, *k*_*e*_	*R*_*tot*_*k*_*e*_, *k*_*on*_, *k*_*off*_	η_*Rtot*/*RC*50_, η_*kon*_, η_*koff*_, η_*Em*_
6	Direct | Dynamic | Sigmoid	*R*_*tot*_, *RC*_50_	*R*_*tot*_/*RC*_50_, *k*_*on*_, *k*_*off*_, *E*_*m*_, *n*	η_*RC*50_, η_*kon*_, η_*koff*_, η_*Em*_, η_*n*_
7	Direct | Dynamic | Indirect	*R*_*tot*_, *k*_*e*_	*R*_*tot*_*k*_*e*_, *k*_*on*_, *k*_*off*_, *k*_*in*_, *k*_*out*_	η_*kon*_, η_*koff*_, η_*kin*_, η_*kout*_, η_*ke*_
8	Direct | Dynamic | Indirect	*R*_*tot*_, *k*_*e*_	*R*_*tot*_*k*_*e*_, *k*_*on*_, *k*_*off*_, *k*_*in*_, *k*_*out*_	η_*kon*_, η_*koff*_, η_*kin*_, η_*kout*_, η_*ke*_
9	Delay | SS | Linear	*R*_*tot*_, *k*_*e*_	*R*_*tot*_*k*_*e*_, *k*_*e*0_, *K*_*d*_	η_*ke*0_, η_*ke*_, η_*Kd*_
10	Delay | SS | Sigmoid	*R*_*tot*_, *RC*_50_	*R*_*tot*_/*RC*_50_, *k*_*e*0_, *k*_*on*_, *k*_*off*_, *E*_*m*_, *n*	η_*ke*0_, η_*RC*50_, η_*Em*_, η_*n*_, η_*Kd*_
11	Delay | SS | Indirect	*R*_*tot*_, *k*_*e*_	*R*_*tot*_*k*_*e*_, *k*_*e*0_, *k*_*in*_, *k*_*out*_, *K*_*d*_	η_*ke*0_, η_*kin*_, η_*kout*_, η_*ke*_, η_*Kd*_
12	Delay | SS | Indirect	*R*_*tot*_, *k*_*e*_	*R*_*tot*_*k*_*e*_, *k*_*e*0_, *k*_*in*_, *k*_*out*_, *K*_*d*_	η_*ke*0_, η_*kin*_, η_*kout*_, η_*ke*_, η_*Kd*_
13	Delay | Dynamic | Linear	*R*_*tot*_, *k*_*e*_	*R*_*tot*_*k*_*e*_, *k*_*e*0_, *k*_*on*_, *k*_*off*_	η_*ke*0_, η_*kon*_, η_*koff*_, η_*ke*_
14	Delay | Dynamic | Sigmoid	*R*_*tot*_, *RC*_50_	*R*_*tot*_/*RC*_50_, *k*_*e*0_, *k*_*on*_, *k*_*off*_, *k*_*in*_, *E*_*m*_, *n*	η_*ke*0_, η_*RC*50_, η_*kon*_, η_*koff*_, η_*Em*_, η_*n*_
15	Delay | Dynamic | Indirect	*R*_*tot*_, *k*_*e*_	*R*_*tot*_*k*_*e*_, *k*_*e*0_, *k*_*on*_, *k*_*off*_, *k*_*in*_, *k*_*out*_	η_*ke*0_, η_*kon*_, η_*koff*_, η_*kin*_, η_*kout*_, η_*ke*_
16	Delay | Dynamic | Indirect	*R*_*tot*_, *k*_*e*_	*R*_*tot*_*k*_*e*_, *k*_*e*0_, *k*_*on*_, *k*_*off*_, *k*_*in*_, *k*_*out*_	η_*ke*0_, η_*kon*_, η_*koff*_, η_*kin*_, η_*kout*_, η_*ke*_

SU, Structurally unidentifiable; SI, Structurally identifiable. ^a^R_tot_ was fixed at 100 when analysing the mixed effects models.

Structurally identifiable and unidentifiable parameters and a suggested reparameterization are provided for the corresponding fixed effects models. Random effects were evaluated for the reparameterized models.

All fixed effects versions of the models were in their original parameterization shown to be structurally unidentifiable. For all of the models, the source of the unidentifiability problem was the parameters *R*_*tot*_ and either *RC*_50_ (Models 2, 6, 10, 14) or *k*_*e*_ (remaining models) (see Table 2). The analysis showed that these parameters are unidentifiable and therefore any numerical estimates of them are effectively meaningless from a biological perspective. Furthermore, it was shown that even though *R*_*tot*_ and *k*_*e*_ or *RC*_50_ are unidentifiable, the product *R*_*tot*_*k*_*e*_ and fraction *R*_*tot*_/*RC*_50_ are globally identifiable. The remaining parameters in the analyzed models were all shown to be globally identifiable. Therefore, three methods may be applied to ensure structurally globally identifiable models: (1) A new parameter may be defined as *R*_*tot*_*k*_*e*_, representing the effect when all targets are bound, and *R*_*tot*_/*RC*_50_, representing the transducer ratio, to replace the unidentifiable parameters. (2) *R*_*tot*_ or (3) *k*_*e*_ and *RC*_50_ may be fixed to known or assumed numerical values. However, this affects the units and interpretation of the non-fixed parameter. For example, *R*_*tot*_ may be fixed at 100%, resulting in changed units for *k*_*e*_ to units per percent bound receptor.

As discussed in Janzén et al. (Under review), if the structural model is structurally globally identifiable, and if the statistical sub-model is structurally globally identifiable, it follows that the mixed-effects model is also structurally globally identifiable. The statistical sub-model for the random effects considered in this paper takes the form of the structurally globally identifiable lognormal distribution. Therefore, the mixed-effects versions of the models in Table 1 are structurally globally identifiable following the reparameterization or fixing of *R*_*tot*_ or *k*_*e*_.

### Simulation study of practical identifiability

In the simulation study, increasing noise, reducing sampling frequency and reducing the number of subjects all led to worse parameter estimation results (Figure 3). At the lowest noise level (column a), the model parameters were well estimated up to a sampling density of Δ*t* = 10, while increasing the sampling interval above this level led to over- and underestimation of *k*_*e*_ and *k*_*on*_ respectively. At the intermediate noise level (column b), similar results were obtained, although problems occurred at smaller sampling intervals. At the highest noise level (column c), the parameter estimation was unsuccessful for all estimation runs except for 100 subjects and 1 min sampling interval. The simulation study shows a trend of decreasing accuracy to estimate the true parameters when the amount and quality of the data decreases. Some of the model parameters vary more than others when the data become worse in terms of noise levels, the number of measurements and the number of subjects. For instance, *k*_*off*_ was estimated reasonably well, except for the very worst case 3c, while the estimates for *k*_*e*_ and *k*_*on*_ are poor in 1a. It can also be seen that the uncertainty in the parameter estimates (standard errors) generally widens with either increased noise, reduced sampling density or reduced number of number subjects. Interestingly, high precision (small standard errors) is in many optimizations acquired despite low accuracy in the parameter estimates.

### Case study: analysing cardiac (side) effects

A case study was conducted in order to exemplify the process of model development, including structural identifiability analysis. Side effects of potential new drugs on the heart must be evaluated by monitoring changes in the duration of specific intervals monitored in the electrocardiogram (ECG), such as the QT interval (defined by the Q and T peaks in the ECG) which corresponds to the duration of the ventricular action potential. The main part of the QT interval constitutes the ventricular repolarization phase, corresponding to the JT interval (defined by the J point and T peak in the ECG), and prolongations are strongly linked to inhibition of the cardiac ion channel hERG (Pollard et al., 2010). In this example, model 10 (Table 1) was applied to link inhibition of the hERG ion channel *in vitro* to prolongation of the JT interval following treatment with the anti-arrhythmic compound and mixed ion channel blocker AZD1305, a proprietary AstraZeneca compound. Model 10 was selected since an identifiable version of this model has been used previously to fit this type of data (Jonker et al., 2005) and following evaluation of additional structures, for example model 2 (without the effect compartment).

#### Methods

Clinical study and PK and QT interval data are described in Parkinson et al. (2013). This phase I study was performed in accordance with the ethical principles of the Declaration of Helsinki and is consistent with the International Conference on Harmonisation (ICH)/Good Clinical Practice. JT intervals were calculated by subtracting QRS from QT. *In vitro* data were acquired from the original data collected by Carlsson et al. (2009). Methods for PKPD model development are detailed in Bergenholm et al. (2016). Baseline variability of JT intervals was minimized applying a circadian rhythm and RR correction models (Chain et al., 2011; Bergenholm et al., 2016). The PK and PD were modeled sequentially, and Model 10 (Table 1) was selected to describe the drug effect. *K*_*d*_ was estimated prior to the PKPD modeling using the *I*_*max*_ model, where the inhibition in % is calculated according to

(9) $$I(C)=100\;*\;C/(IC_{50}+C)$$

where *IC*_50_ corresponds to the drug concentration resulting in 50% inhibition, substituting *K*_*d*_ in Model 10. Parameter estimations were performed using the stochastic approximation expectation maximization (SAEM) algorithm as implemented in Monolix 4.3.2 (Lixoft, 2012).

#### Results

**Figure 4:**
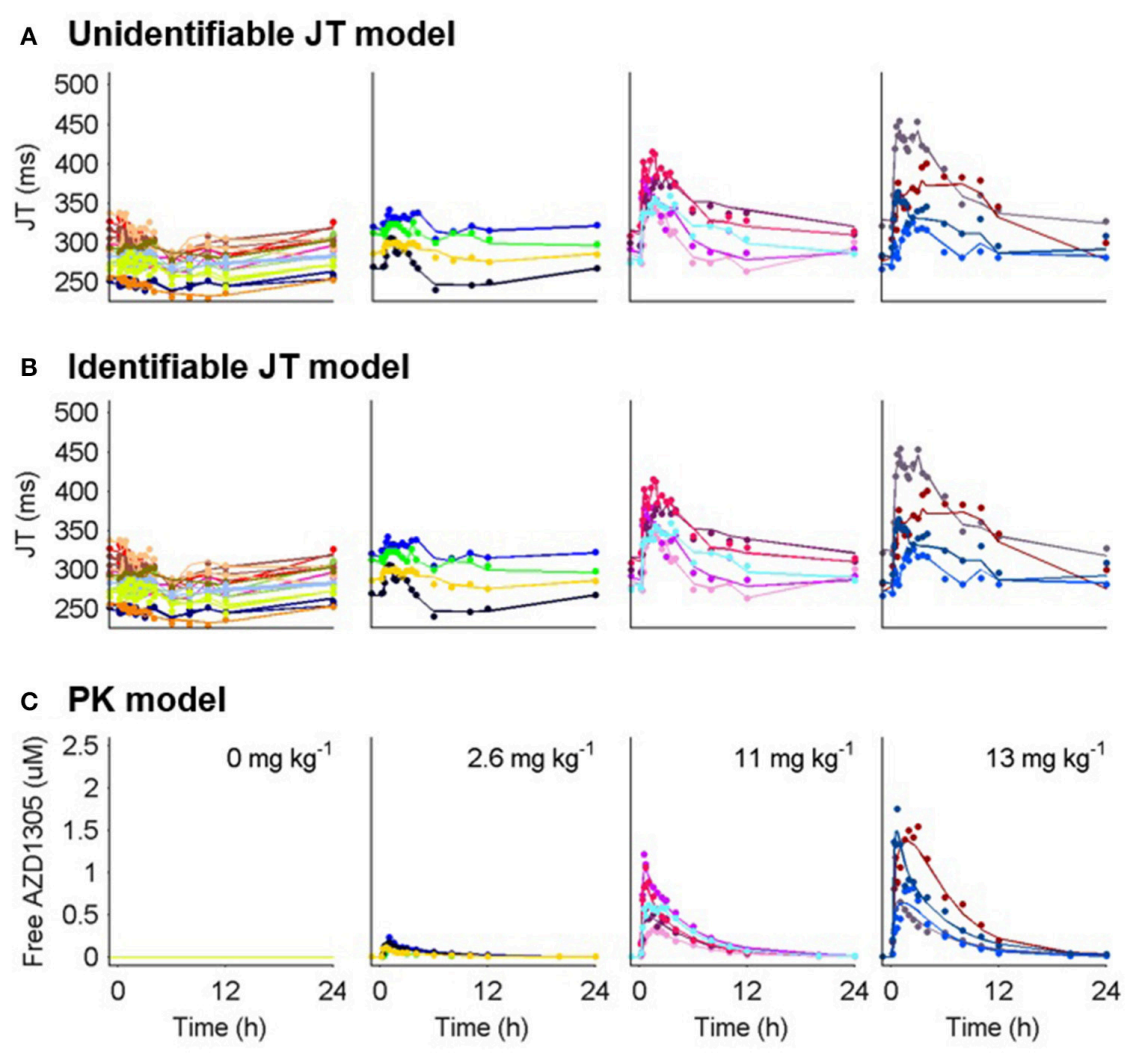
**PK and JT interval data (markers) and model predictions (lines) for humans treated with placebo and 3 selected doses of AZD1305**. **(A)** Model predictions by the unidentifiable JT model. **(B)** Model predictions by the identifiable JT model. **(C)** Individual PK model parameters predicting the PK in each subject were used to drive the PD response. Individual subjects are separated by color.

The estimated *IC*_50_ of hERG was 0.37 ± 0.04 μM with between cell variability of 0.19 ± 0.09 μM. Fitting all parameters of the operational model led to high uncertainty and correlation between *R*_*tot*_ and *RC*_50_ (Table 3). Structural identifiability analysis of this model showed that only the fraction *R*_*tot*_/*RC*_50_ is identifiable (see Table 2) and the model was therefore reparameterized with τ = *R*_*tot*_/*RC*_50_, resulting in a structurally identifiable model. Estimation of the reduced model resulted in similar parameter values for all of the identifiable parameters, similar goodness of fit values and residuals and good precision in the population estimate of τ (Table 3). The fits to the generated data can be seen in Figure 4.

**Table 3:** **Estimated parameter values for the original and re-parameterized Model 10 fitted to AZD1305 PK-hERG-JT interval data**.

**Parameter**	**Unit**	**Unidentifiable model**		**Identifiable model**	
		**Estimate (SE)**	**BSV % (SE)**	**Estimate (SE)**	**BSV % (SE)**
*E*_*m*_	ms	172 (23.9)	18.7 (9.09)	162 (18.9)	20.6 (7.67)
*RC*_50_	μM	0.753 (173)	13.3 (15300)	–	–
*n*		2.02 (0.24)	35.1 (7.5)	2.1 (0.219)	36.4 (7.69)
*R*_*tot*_	μM	1.1 (252)	13.2 (15400)	–	–
τ		–	–	1.55 (0.163)	15.2 (8.17)
*IC*_50_	μM	0.37 (fixed)	0.19 (fixed)	0.37 (fixed)	0.19 (fixed)
*k*_*e*0_	h^−1^	9.37 (2.96)	125 (24)	9.42 (2.91)	123 (23.4)
*Residuals*	ms	6.64 (0.155)	–	6.64 (0.155)	–
−*2LL*		7662		7670	

SE, Standard error; BSV, Between-subject variability; −2LL, −2 LogLikelihood.

#### Discussion

Both the full and reparametererized versions of model 10 described the data well. However, standard errors and correlations of *R*_*tot*_ and *RC*_50_ correctly indicated identifiability issues with the former. The estimated parameters were converted to the traditional *E*_*max*_ and *EC*_50_ parameters, which describe the maximal effect and the drug concentration at half-maximum effect respectively. *E*_*max*_ and *EC*_50_ were calculated according to

(10) $$E_{max}=\frac{E_{m}\tau^{n}}{1+\tau^{n}}$$

(11) $$EC_{50}=\frac{IC_{50}}{{\left(2+\tau^{n}\right)}^{1/n}-1}$$

and resulted in an estimated *E*_*max*_ of 117 ms and 116 ms and *EC*_50_ of 0.36 and 0.35 μM respectively for the full and reparameterized models. This highlights that identifiable parts of a structurally unidentifiable model are still informative. The estimated *E*_*max*_ is similar to that in previous hERG-QT modeling of dofetilide (Jonker et al., 2005), while the estimated hERG block at 10 ms JT prolongation was slightly higher (18 vs. 9%). This may be caused by AZD1305-induced calcium block (Carlsson et al., 2009), as the calcium current depolarizes the cardiac cells (Amin et al., 2010), counter-acting the repolarization by hERG. The structural identifiability analysis showed that two model parameters could not be estimated. This led to model reduction. Performing this analysis prior to parameter estimation ensures the theoretical possibility of estimating all parameters in the model. Estimating the parameters of the unidentifiable model could have been avoided, reducing the number of iterations in the optimization. Also, ensuring structural identifiability improves confidence in the biological interpretation of the estimated parameter values.

## Discussion

Unidentifiability issues can cause many different types of problems if not mitigated when models are used to quantify, predict and understand the effects of potential drugs. Most importantly, the biological/physiological interpretations of structurally or practically unidentifiable parameters are not valid. This may lead to wrong conclusions, for example when unknowingly comparing unidentifiable parameters to rate candidate drugs or for comparison with competitors. Also, any predictions based on the profiles of unmeasured states of the system may be meaningless if the parameters directly or indirectly related to those states are unidentifiable. For example, if the effect of interest in a toxicity or efficacy study depends on the concentration in a compartment for which the profile is linked to structurally unidentifiable parameters, it may be impossible to separate the distribution to this compartment and the drug effect. Unidentifiability issues may also cause technical problems, as the parameter estimation step may take a very long time, or fail (crash), if a structurally unidentifiable model is used (depending on what form of optimization routine is used).

We have investigated the structural identifiability of 16 fundamental pharmacodynamic models and identified parameterizations that are structurally identifiable both for fixed effects- and mixed-effects- versions of the models, as summarized in Table 2. For all of the investigated models, the total amount of receptor in the system was fixed (to e.g., 1 or 100%) in order to achieve structural identifiability. This implies that some parameters for the “signal transduction” are relative. For example, the units of a proportional signal transduction are effect units per fraction bound/inhibited receptor if *R*_*tot*_ is fixed to 1. This analysis shows that given sufficient data quality, it is, in theory, possible to distinguish between different sources of delay from the data. Thus, it is possible to differentiate delays that are compound-specific (e.g., distribution, drug-receptor binding kinetics) from delays that are system-specific (e.g., turnover of receptors) to compare compounds and simulate untested systems. The investigated models have been used successfully and repeatedly in practice (Ploeger et al., 2009; Peletier and Gabrielsson, 2012), and our results confirm the general assumption of structural identifiability. This provides confidence in the theoretical soundness of using these models.

**Figure 5:**
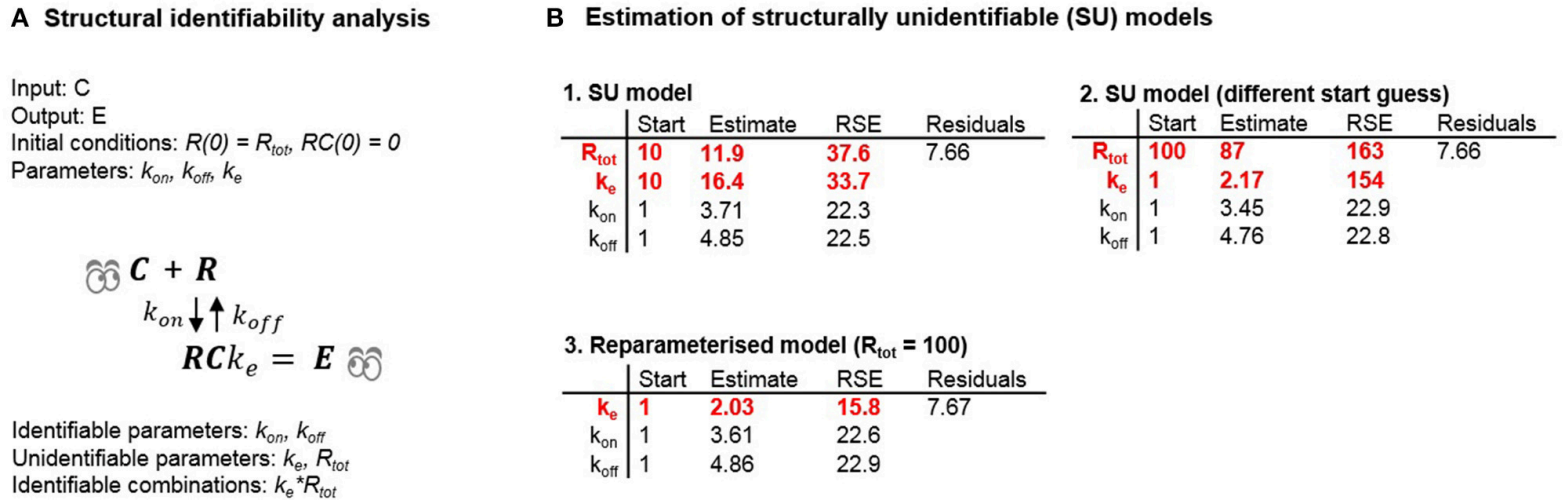
**(A)** Results of the structural identifiability analysis of Model 5. **(B)** Optimization results following estimation of unidentifiable and identifiable versions of Model 5 using example data.

Next, we estimated parameters of the unidentifiable and reparameterized versions of Model 5 (Tables 1, 2) in to investigate the possible consequences of estimating unidentifiable models (Figure 5). Three separate runs of parameter estimation were performed. Parameters in the unidentifiable version of the model were estimated in two different runs using different initial estimates. For the third parameter estimation run, the model was reparameterized following insights from the structural identifiability analysis.

Investigating the estimated parameters shows that standard errors of unidentifiable parameters differ significantly between the two estimation runs, and are larger than the standard error of the product of the parameters. For one of the estimation runs the magnitude of the standard errors (37.6 and 33.7%) did not clearly indicate a structural identifiability problem. In the second estimation run the standard errors (163 and 154%) did indicate a structural identifiability problem. However, for both estimation runs the estimated correlations between *R*_*tot*_ and *k*_*e*_ were −0.9 and −0.99 respectively, indicating a potential structural identifiability problem in both cases. Alternatively, analysing the models using the profile likelihood approach (Raue et al., 2009) would also potentially indicate a problem with structural and practical identifiability. Although estimation of an unidentifiable model in theory should lead to infinitely large uncertainty for the structurally unidentifiable parameters due to a flat likelihood function in the directions representing those parameters, this did not happen in practice. The reason why this did not happen can be explained by measurement and numerical noise. In real-world problems, the likelihood function is never completely flat which introduces false local minima where the optimization routine may become “stuck” depending on the initial guesses used for the model parameters and the optimization algorithm itself. This example shows the potential danger of using practical identifiability analysis as a tool to deduce structural identifiability. For the first set of initial guesses for the parameters, the reported RSE-values are unreasonably high indicating a structural identifiability issue. However, the RSE-values reported using a different set of initial guesses for the model parameters do not indicate that there is any structural identifiability problem. The results of these estimations were used to draw some general conclusions. These are as follows:

- Different initial guesses of the model parameters may lead to different estimates of structurally unidentifiable parameters.
- Large standard errors may indicate that a parameter is structurally (or practically) unidentifiable but **unidentifiable parameters may also appear well-determined**.
- Reparameterizing the structurally unidentifiable model to become identifiable leads to similar residuals (and likelihood) and improved parameter precision of the new parameter(s).
- Identifiable parameters can still be well-determined when other parameters are unidentifiable.

Similar findings regarding masking of structural unidentifiability, i.e., estimation of seemingly reasonable RSE-values of structurally unidentifiable parameters, has been reported in the conference contribution (Aoki et al., 2015) and in the follow-up paper (Aoki et al., 2014). These findings were reported using NONMEM, rather than Monolix, which indicates that estimation of misleading RSE-values under structural unidentifiability conditions is not a software specific issue but instead a general numerical computational instability issue. In these two publications, a numerical approach called preconditioning is suggested. In short, this approach involves reparametrization of the model in such a way so that the subsequent numerical computations of the RSE-values reportedly becomes more stable and thus more reliable under structurally unidentifiable conditions.

It is important to remember that having a structurally identifiable model is only a prerequisite for successful parameter estimation. In other words, that parameters are identifiable with ideal data (continuous, noise-free data from an infinite number of subjects in the mixed effects model case) does not guarantee that they will be practically identifiable with a finite number of noisy data points from a finite number of subjects.

The effects of practical identifiability were investigated in a simulation study, where the quality of the data was varied from good to worse, but the structural model was known to be identifiable (Model 13). Conclusions from this example are that:

- A structurally identifiable model does not guarantee reliable parameter estimates.
- Data must contain information over relevant time scales for the investigated system.
- Noise levels, sampling density and the number of subjects (mixed-effects models) are all important in order to be able to estimate parameters with reasonably high precision.

When the data do not contain information on the time scale of the rate parameters in the system, the model should be reduced to only account for effects over the relevant time scales. This applies even when all parameters are structurally identifiable.

## Conclusions

Parameter identifiability should be investigated to ensure both structural and practical identifiability. Our work confirms the structural identifiability of a set of fundamental pharmacodynamic models, and provides examples of estimation results with unidentifiable models. The investigated models have been proven to have a sound theoretical basis in terms of structural identifiability and thus are reliable in this respect. This in turn increases the reliability of using such models in clinical pharmacology and therapeutics.

## Author contributions

All authors participated in the design of the research study. DJ performed the structural identifiability analyses. LB and DJ performed the practical identifiability analyses. LB conducted the analyses for the case study. All authors participated in analysing the results. All authors participated in writing the manuscript.

### Conflict of interest statement

The authors declare that the research was conducted in the absence of any commercial or financial relationships that could be construed as a potential conflict of interest.

## References

[B1] Amin A. S. Tan H. L. Wilde A. A. (2010). Cardiac ion channels in health and disease. Heart Rhythm 7, 117–126. 10.1016/j.hrthm.2009.08.005 19875343

[B2] Aoki Y. Nordgren R. Hooker A. C. (2014). Preconditioning of nonlinear mixed effects models for stabilisation of variance-covariance matrix computations. AAPS J. 18, 505–518. 10.1208/s12248-016-9866-5 26857397 PMC4779107

[B3] Aoki Y. Nordgren R. Hooker A. C. (2015). Preconditioning of Nonlinear Mixed Effect Models for Stabilization of the Covariance Matrix Computation. Available online at: http://www.page-meeting.org/?abstract=3586 10.1208/s12248-016-9866-5 PMC4779107 26857397

[B4] Bearup D. J. Evans N. D. Chappell M. J. (2013). The input-output relationship approach to structural identifiability analysis. Comput. Methods Prog. Biomed. 109, 171–181. 10.1016/j.cmpb.2012.10.012 23228562

[B5] Bellman R. Åström K. J. (1970). On structural identifiability. Math. Biosci. 7, 329–339. 10.1016/0025-5564(70)90132-X849680

[B6] Bergenholm L. Collins T. Evans N. D. Chappell M. J. Parkinson J. (2016). PKPD modelling of PR and QRS intervals in conscious dogs using standard safety pharmacology data. J. Pharm. Toxicol. Methods 79, 34–44. 10.1016/j.vascn.2016.01.002 26780675

[B7] Black J. W. Leff P. (1983). Operational models of pharmacological agonism. Proc. R. Soc. B Biol. Sci. 220, 141–162. 10.1098/rspb.1983.0093 6141562

[B8] Carlsson L. Andersson B. Linhardt G. Löfberg L. (2009). Assessment of the ion channel-blocking profile of the novel combined ion channel blocker AZD1305 and its proarrhythmic potential versus dofetilide in the methoxamine-sensitized rabbit *in vivo*. J. Cardiovasc. Pharmacol. 54, 82–89. 10.1097/FJC.0b013e3181ac62c9 19528812

[B9] Chain A. S. Krudys K. M. Danhof M. Della Pasqua O. (2011). Assessing the probability of drug-induced QTc-interval prolongation during clinical drug development. Clin. Pharmacol. Ther. 90, 867–875. 10.1038/clpt.2011.202 22048226

[B10] Chappell M. J. (1996). Structural identifiability of models characterizing saturable binding: comparison of pseudo-steady-state and non-pseudo-steady-state model formulations. Math. Biosci. 133, 1–20. 10.1016/0025-5564(95)00064-X 8868570

[B11] Cheung S. Y. Yates J. W. Aarons L. (2013). The design and analysis of parallel experiments to produce structurally identifiable models. J. Pharmacokinet. Pharmacodyn. 40, 93–100. 10.1007/s10928-012-9291-z 23300030

[B12] Danhof M. de Jongh J. De Lange E. C. Della Pasqua O. Ploeger B. A. Voskuyl R. A. (2007). Mechanism-based pharmacokinetic-pharmacodynamic modeling: biophase distribution, receptor theory, and dynamical systems analysis. Ann. Rev. Pharmacol. Toxicol. 47, 357–400. 10.1146/annurev.pharmtox.47.120505.105154 17067280

[B13] Eudy R. J. Riggs M. M. Gastonguay M. R. (2015). A priori identifiability of target-mediated drug disposition models and approximations. AAPS J. 17, 1280–1284. 10.1208/s12248-015-9795-8 26077506 PMC4540726

[B14] Evans N. Moyse H. Lowe D. Briggs D. Higgins R. Mitchell D. (2013). Structural identifiability of surface binding reactions involving heterogeneous analyte : application to surface plasmon resonance experiments. Automatica 49, 48–57. 10.1016/j.automatica.2012.09.015

[B15] Evans N. D. Errington R. J. Shelley M. Feeney G. P. Chapman M. J. Godfrey K. R.. (2004). A mathematical model for the *in vitro* kinetics of the anti-cancer agent topotecan. Math. Biosci. 189, 185–217. 10.1016/j.mbs.2004.01.007 15094319

[B16] Evans N. D. Godfrey K. R. Chapman M. J. Chappell M. J. Aarons L. Dufull S. D. (2001). An identifiability analysis of a parent-metabolite pharmacokinetic model for ivabradine. J. Pharmacokinet. Pharmacodyn. 28, 93–105. 10.1023/A:1011521819898 11253617

[B17] Gabrielsson J. Fjellström O. Ulander J. Rowley M. Van Der Graaf P. H. (2011). Pharmacodynamic-pharmacokinetic integration as a guide to medicinal chemistry. Curr. Top. Med. Chem. 11, 404–418. 10.2174/156802611794480864 21320067

[B18] Janzén D. L. I. Jirstrand M. Chappell M. J. Evans N. D. (2016). Three novel approaches to structural identifiability analysis in mixed-effects models. Comput. Methods Prog. Biomed. 10.1016/j.cmpb.2016.04.024. [Epub ahead of print]. 27181677

[B19] Jonker D. M. Kenna L. A. Leishman D. Wallis R. Milligan P. A. Jonsson E. N. (2005). A pharmacokinetic-pharmacodynamic model for the quantitative prediction of dofetilide clinical QT prolongation from human ether-a-go-go-related gene current inhibition data. Clin. Pharmacol. Therapeut. 77, 572–582. 10.1016/j.clpt.2005.02.004 15961988

[B20] Karlsson J. Anguelova M. Jirstrand M. (2012). An efficient method for structural identifiability analysis of large dynamic systems, in 16th IFAC Symposium on system identification (Brussels), 941–946.

[B21] Lixoft (2012). Monolix 4.3.2.

[B22] Mager D. E. Jusko W. J. (2001). General pharmacokinetic model for drugs exhibiting target-mediated drug disposition. J. Pharmacokinet. Pharmacodyn. 28, 507–532. 10.1023/A:1014414520282 11999290

[B23] Parkinson J. Visser S. A. Jarvis P. Pollard C. Valentin J. P. Yates J. W.. (2013). Translational pharmacokinetic-pharmacodynamic modeling of QTc effects in dog and human. J. Pharmacol. Toxicol. Methods 68, 357–366. 10.1016/j.vascn.2013.03.007 23567074

[B24] Peletier L. A. Gabrielsson J. (2012). Dynamics of target-mediated drug disposition: characteristic profiles and parameter identification. J. Pharmacokinet. Pharmacodyn. 39, 429–451. 10.1007/s10928-012-9260-6 22851162 PMC3446204

[B25] Ploeger B. A. van der Graaf P. H. Danhof M. (2009). Incorporating receptor theory in mechanism-based pharmacokinetic-pharmacodynamic (PK-PD) modeling. Drug Metab. Pharmacokinet. 24, 3–15. 10.2133/dmpk.24.3 19252332

[B26] Pollard C. E. Abi Gerges N. Bridgland-Taylor M. H. Easter A. Hammond T. G. Valentin J. P. (2010). An introduction to QT interval prolongation and non-clinical approaches to assessing and reducing risk. Br. J. Pharmacol. 159, 12–21. 10.1111/j.1476-5381.2009.00207.x 20141516 PMC2823347

[B27] Raue A. Karlsson J. Saccomani M. P. Jirstrand M. Timmes J. (2014). Comparison of approaches for parameter identifiability analysis of biological systems. Bioinformatics 30, 1440–1448. 10.1093/bioinformatics/btu006 24463185

[B28] Raue A. Kreutz C. Maiwald T. Bachmann J. Schilling M. Klingmuller U.. (2009). Structural and practical identifiability analysis of partially observed dynamical models by exploiting the profile likelihood. Bioinformatics 25, 1923–1929. 10.1093/bioinformatics/btp358 19505944

[B29] Sheiner L. B. Stanski D. R. Vozeh S. Miller R. D. Ham J. (1979). Simultaneous modeling of pharmacokinetics and pharmacodynamics: application to d-tubocurarine. Clin. Pharmacol. Therapeut. 25, 358–371. 10.1002/cpt1979253358 761446

[B30] The MathWorks Inc. (2016). The MathWorks, Inc. Natick, MA: Matlab 2013b.

