# Supplementary material for: Parameter Identifiability of Fundamental Pharmacodynamic Models
Source: Front Physiol. 2016 Dec 5;7:590. doi: 10.3389/fphys.2016.00590 (PMC5136565; doi:10.3389/fphys.2016.00590)
Supplement: Supplementary file 1 [file DataSheet1.pdf]

Supplementary materials

Identifiability analysis of fixed-effects model

Example structural identifiability analysis of the fixed effects model version of Model 13, a dynamic receptor binding model with an effect compartment and a proportional drug effect. The structure of the model is given by

$$\begin{aligned}\dot{C_e} &= k_{e0}(C_p - C_e)\\ \dot{RC} &= k_{on}(R_{tot} - RC)C_e - k_{off}RC\\ E &= k_e RC\end{aligned}$$

where E is the observed effect, the unknown parameter vector $\boldsymbol{\theta} = (k_{e0}, k_e, k_{on}, k_{off})$, C_p is the plasma drug concentration, here treated as a known input signal, C_e is a state representing the concentration in the hypothetical effect compartment, RC is the drug-receptor complex, and R_{tot} is the total number of receptor, which is fixed. The approach chosen to study structural identifiability here is the input-output approach [4]. By iteratively differentiating the output signal and eliminating the state variables the model can be rewritten on the following input-output form

$$\begin{aligned}&- {R_{tot}}^2 C_p k_e^2 k_{e0} k_{on} - 2\, R_{tot}\, C_p k_e k_{e0} k_{on} E + R_{tot}\, k_e k_{e0} k_{off} E -\\ &C_p k_{e0} k_{on} E^2 + R_{tot}\, k_e k_{e0} \dot{E} + R_{tot}\, k_e k_{off} \dot{E} + k_{e0} k_{off} E^2 +\\ &R_{tot}\, k_e \ddot{E} + k_{e0} E\dot{E} + E\ddot{E} - \dot{E}^2 = 0.\end{aligned}$$

The structural identifiability of a model can be studied by considering the coefficients in the input-output form of the model. By introducing an alternative parameter vector $\bar{\boldsymbol{\theta}}$ and collecting the coefficients in the input-output form as

$$\sum_{k=1}^{l} c_k(\boldsymbol{\theta}, \bar{\boldsymbol{\theta}})\phi_k(E(t,\boldsymbol{\theta}), \dot{E}(t,\boldsymbol{\theta}), \ddot{E}(t,\boldsymbol{\theta}), \dots) = 0$$

we can study the structural identifiability by solving the following system of algebraic equations

$$\begin{aligned}c_1(\boldsymbol{\theta}, \bar{\boldsymbol{\theta}}) &= k_{e0} - \bar{k}_{e0} = 0\\ c_2(\boldsymbol{\theta}, \boldsymbol{\theta}) &= R_{tot}k_e - R_{tot}^2 u k_e^2 k_{e0} k_{on} - (\bar{R}_{tot}\bar{k}_e - \bar{R}_{tot}^2 u \bar{k}_e^2 \bar{k}_{e0}\bar{k}_{on}) = 0\\ c_3(\boldsymbol{\theta}, \bar{\boldsymbol{\theta}}) &= R_{tot}(k_e k_{e0} + k_e k_{off}) - \bar{R}_{tot}(\bar{k}_e \bar{k}_{e0} + \bar{k}_e \bar{k}_{off}) = 0\\ c_4(\boldsymbol{\theta}, \bar{\boldsymbol{\theta}}) &= -u k_{e0} k_{on} + k_{e0} k_{off} - (-u\bar{k}_{e0}\bar{k}_{on} + \bar{k}_{e0}\bar{k}_{off}) = 0\\ c_5(\boldsymbol{\theta}, \bar{\boldsymbol{\theta}}) &= -R_{tot} u k_e k_{e0} k_{on} + R_{tot} k_e k_{e0} k_{off} -\\ &\quad (-\bar{R}_{tot} u \bar{k}_e \bar{k}_{e0} \bar{k}_{on} + \bar{R}_{tot} \bar{k}_e \bar{k}_{e0} \bar{k}_{off}) = 0.\end{aligned}$$

By visual inspection it can be seen that k_e and R_{tot} always appear together as a product. Because of this it can be concluded that the model is structurally unidentifiable. The parameter R_{tot} is therefore fixed, i.e., $R_{tot} = \bar{R}_{tot}$. From (15) we have that $k_{e0} = \bar{k}_{e0}$. Equation (18) can then be rewritten as

$$-uk_{on} + k_{off} = -u\bar{k}_{on} + \bar{k}_{off}.$$

Again, by using $k_{e0} = \bar{k}_{e0}$ and that generically $k_{e0} \neq 0$, equation (19) can be rewritten as

$$k_e(-uk_{on} + k_{off}) = \bar{k}_e(-u\bar{k}_{on} + \bar{k}_{off}).$$

Combining (20) and (21) gives $k_e = \bar{k}_e$. From here it is easy to see, using previous results, from (16) and (17) that $k_{on} = \bar{k}_{on}$ and $k_{off} = \bar{k}_{off}$. In other words, this particular system has one solution $\boldsymbol{\theta} = \bar{\boldsymbol{\theta}}$ which means that the model is structurally globally identifiable.

Identifiability analysis of mixed-effects model

Example structural identifiability analysis of the mixed effects model version of Model 13 with R_{tot} fixed. The functions of the random variables are

$$\begin{aligned}
Z_1 &= k_{e0}e^{\eta_{ke0}} \\
Z_2 &= R_{tot}k_e e^{\eta_{ke}}(1 - R_{tot}uk_e e^{\eta_{ke}}k_{e0}e^{\eta_{keo}}k_{on}e^{\eta_{kon}}) \\
Z_3 &= R_{tot}(k_e e^{\eta_{ke}}k_{e0}e^{\eta_{e0}} + k_e e^{\eta_{ke}}k_{off}e^{\eta_{koff}}) \\
Z_4 &= R_{tot}(-uk_e e^{\eta_{ke}}k_{e0}e^{\eta_{ke0}}k_{on}e^{\eta_{kon}} + k_e e^{\eta_{ke}}k_{e0}e^{\eta_{ke0}}k_{on}e^{\eta_{koff}}) \\
Z_5 &= R_{tot}k_e e^{\eta_{ke}}k_{e0}e^{\eta_{ke0}}(k_{on}e^{\eta_{kon}} + k_{off}e^{\eta_{koff}}).
\end{aligned}$$

From these functions of random variables the statistical moments are computed, denoted $\mathbb{E}[Z_i^m]$ where $i = 1, 2, 3, 4, 5$ with the $m : th$ order of statistical moment. By introducing an alternative population vector and covariance matrix and equating the first two statistical moments of the functions of random variables the following expression is obtained

$$\begin{aligned}
\mathbb{E}[Z_1(\boldsymbol{\theta}, \boldsymbol{\eta})] &= \mathbb{E}[Z_1(\bar{\boldsymbol{\theta}}, \bar{\boldsymbol{\eta}})] \\
\mathbb{E}[Z_1^2(\boldsymbol{\theta}, \boldsymbol{\eta})] &= \mathbb{E}[Z_1^2(\bar{\boldsymbol{\theta}}, \bar{\boldsymbol{\eta}})] \\
\mathbb{E}[Z_2(\boldsymbol{\theta}, \boldsymbol{\eta})] &= \mathbb{E}[Z_2(\bar{\boldsymbol{\theta}}, \bar{\boldsymbol{\eta}})] \\
\mathbb{E}[Z_2^2(\boldsymbol{\theta}, \boldsymbol{\eta})] &= \mathbb{E}[Z_2^2(\bar{\boldsymbol{\theta}}, \bar{\boldsymbol{\eta}})] \\
\mathbb{E}[Z_3(\boldsymbol{\theta}, \boldsymbol{\eta})] &= \mathbb{E}[Z_3(\bar{\boldsymbol{\theta}}, \bar{\boldsymbol{\eta}})] \\
\mathbb{E}[Z_3^2(\boldsymbol{\theta}, \boldsymbol{\eta})] &= \mathbb{E}[Z_3^2(\bar{\boldsymbol{\theta}}, \bar{\boldsymbol{\eta}})] \\
\mathbb{E}[Z_4(\boldsymbol{\theta}, \boldsymbol{\eta})] &= \mathbb{E}[Z_4(\bar{\boldsymbol{\theta}}, \bar{\boldsymbol{\eta}})] \\
\mathbb{E}[Z_4^2(\boldsymbol{\theta}, \boldsymbol{\eta})] &= \mathbb{E}[Z_4^2(\bar{\boldsymbol{\theta}}, \bar{\boldsymbol{\eta}})] \\
\mathbb{E}[Z_5(\boldsymbol{\theta}, \boldsymbol{\eta})] &= \mathbb{E}[Z_5(\bar{\boldsymbol{\theta}}, \bar{\boldsymbol{\eta}})] \\
\mathbb{E}[Z_5^2(\boldsymbol{\theta}, \boldsymbol{\eta})] &= \mathbb{E}[Z_5^2(\bar{\boldsymbol{\theta}}, \bar{\boldsymbol{\eta}})]
\end{aligned}$$

By solving the equation system above for both $\boldsymbol{\theta}, \boldsymbol{\eta}$ the identifiability of the mixed-effects version of the model can be studied. The symbolic analysis was done in Mathematica and the expression have here been omitted due to their large size. From the analysis it could be concluded that there was only only solution to the equation system above, namely

$$\{\boldsymbol{\theta}, \boldsymbol{\eta}\} = \{\bar{\boldsymbol{\theta}}, \bar{\boldsymbol{\eta}}\}.$$

The mixed-effects model is therefore structurally globally identifiable with the parameter R_{tot} fixed.

Below is the maple code used for generation of the input-output form which is based on Forsman approach outlined in [16]. The MAPLE version used was MAPLE 15.

```
with(LinearAlgebra) : with(Groebner) :
```

Forsman Code

```
lieDer :=proc(H, F, vars)
  local V:
  V := map((a, b)→diff(b, a), vars, H) :
  DotProduct(Vector(F), Vector(V), conjugate= false)
 end:

listLieDer := proc(H, F, k)
  local L, i, tmp, N, vars :
  L := [y[0]−H] : tmp := H :
  N := nops(F) ;
  vars := [seq(x[t], t = 1 .. N)] ;
  for i to k do
   tmp := lieDer(tmp, F, vars) :
   L := [op(L), y[i]-tmp]
 od;
end:
xlistLieDer := proc(H, F, k, uvars)
  local L, i, f, h, tmp, N, var, vars, duvars ;
  N := nops(F) ;
  f := F ;
  h := H ;
  for var in uvars do
```

```
    f := subs(var = var[0], f) ;
    h := subs(var = var[0], h) ;
  od;
  duvars := [ ] ;
  vars := [seq(x[t], t = 1 ..N)] ;
  for var in uvars do
    vars := [op(vars), seq(var[i], i = 0 ..10)] ;
    duvars := [op(duvars), seq(var[i], i = 1 ..11)] ;
  od;
  f := [op(f), op(duvars)] ;
  L := [y[0]-h]; tmp := h ;
  i := 1 ;
  for i to k do
    tmp := lieDer(tmp, f, vars) ;
    L := [op(L), y[i]-tmp] ;
   od;
 end:

iorel := proc(f, h, uvars)
  local n, L :
  n := nops(f) :
  if _params['uvars'] = NULL then
    L := listLieDer(h, f, n) :
 else
   L := xlistLieDer(h, f, n, uvars) :
 fi;
 L := map(expand, numer(L)) :
 UnivariatePolynomial(y[n], L, [seq(x[t], t = 1 ..n), y[n]]) ;
end:
```

Model 2 (sigmoid, augmented, m is here the parameter in the exponent in the original system.)

Defining the system as:

$$F := \left[-x[1], \frac{m \cdot k[d] \cdot x[2]}{k[d] + x[1]} \right]$$

$$\left[-x_1, \frac{m\, k_d\, x_2}{k_d + x_1} \right]$$

$$H := \frac{E[maxx]}{x[2] \cdot RC[50] + 1} \;;$$

$$\frac{E_{maxx}}{x_2\, RC_{50} + 1}$$

$outptEqn := iorel(\mathrm{F}, \mathrm{H})$

$$-m\, y_1\, y_0\, E_{maxx} + m\, E_{maxx}\, y_0\, y_2 - m\, E_{maxx}\, y_1^2 + m\, y_1\, y_0^2 - m\, y_2\, y_0^2 + 2\, m\, y_0\, y_1^2 - E_{maxx}\, y_1^2$$

Collecting the coefficients and setting up expression with alternative parameter vector

$uA := \{coeffs(collect(outptEqn, [y[0], y[1], y[2]], 'distributed'), [y[0], y[1], y[2]])\}$

$$\{m, m\, E_{maxx}, -m, 2\, m, -m\, E_{maxx}, -m\, E_{maxx} - E_{maxx}\}$$

$uB := eval(uA, [m = mb, E[maxx] = Eb[maxx], RC[50] = RCb[50], k[d] = kb[d]])$

$$\{mb, mb\, Eb_{maxx}, -mb, 2\, mb, -mb\, Eb_{maxx}, -mb\, Eb_{maxx} - Eb_{maxx}\}$$

$eqns := convert(uA, list) - convert(uB, list)$

$$[-mb + m, -mb\, Eb_{maxx} + m\, E_{maxx}, mb - m, -2\, mb + 2\, m, mb\, Eb_{maxx} - m\, E_{maxx}, mb\, Eb_{maxx} + Eb_{maxx} - m\, E_{maxx} - E_{maxx}]$$

Setting up the equations for the inital conditions

$n := 2 :$

Calculating the derivative of output function y, then evaluating the expressions for the initial conditions

$icsnoteval := eval(\ -1 * listLieDer(H, F, n-1), [seq(y[i] = 0, i = 0 .. n-1)]\ )$

$$\left[\frac{E_{maxx}}{x_2\, RC_{50} + 1}, -\frac{m\, k_d\, x_2\, E_{maxx}\, RC_{50}}{(k_d + x_1)\, (x_2\, RC_{50} + 1)^2}\right]$$

$icseval := eval(icsnoteval, \{x[1] = Dose, x[2] = a0\})$

$$\left[\frac{E_{maxx}}{a0\, RC_{50} + 1}, -\frac{m\, k_d\, a0\, E_{maxx}\, RC_{50}}{(k_d + Dose)\, (a0\, RC_{50} + 1)^2}\right]$$

Introducing alternative parameter vector and finalizing the expression for the initial conditions

$icsA := Vector(icseval);$

$icsB := eval(icsA, [m = mb, E[maxx] = Eb[maxx], RC[50] = RCb[50], k[d] = kb[d]]);$

$$\begin{bmatrix} \dfrac{E_{maxx}}{a0\, RC_{50} + 1} \\ -\dfrac{m\, k_d\, a0\, E_{maxx}\, RC_{50}}{(k_d + Dose)\, (a0\, RC_{50} + 1)^2} \end{bmatrix}$$

$ics := [icsA(1) - icsB(1), icsA(2) - icsB(2)]$

$$\left[\frac{E_{maxx}}{a0\, RC_{50} + 1} - \frac{Eb_{maxx}}{a0\, RCb_{50} + 1}, -\frac{m\, k_d\, a0\, E_{maxx}\, RC_{50}}{(k_d + Dose)\, (a0\, RC_{50} + 1)^2} + \frac{mb\, kb_d\, a0\, Eb_{maxx}\, RCb_{50}}{(kb_d + Dose)\, (a0\, RCb_{50} + 1)^2}\right]$$

$solve([op(ics), op(eqns)], [m, E[maxx], RC[50], k[d]])$

$$[[m = mb, E_{maxx} = Eb_{maxx}, RC_{50} = RCb_{50}, k_d = kb_d]]$$

Summary: The model is globally identifiable.
